# Development of Alkaline-Activated Self-Leveling Hybrid Mortar Ash-Based Composites

**DOI:** 10.3390/ma11101829

**Published:** 2018-09-26

**Authors:** Luís Urbano Durlo Tambara Júnior, Malik Cheriaf, Janaíde Cavalcante Rocha

**Affiliations:** Laboratory of Waste Valorization and Sustainable Materials (ValoRes), Department of Civil Engineering, PPGEC, Federal University of Santa Catarina (USFC), Campus Trindade, 88040-900 Florianópolis, SC, Brazil; luis.tambara@posgrad.ufsc.br (L.U.D.T.J.); malik.cheriaf@gmail.com (M.C.)

**Keywords:** hybrid cements, self-levelling mortar, ashes, alkali-activation

## Abstract

This study investigated the reactivity properties of self-leveling hybrid alkali-activated cements, such as ordinary Portland cement (OPC) and its residual precursors, coal bottom ash (BA), and rice husk ash (RHA). Due to the relatively low reactivity of BA, binary mixes were produced with OPC using contents of 2.5–30% in the treated BA samples. Furthermore, ternary mixes were prepared in proportions of 25%, 50%, and 75% with RHA as a replacement material for the OPC (mix with 90%:10% BA:OPC). For all of the mixes the spreading behaviors were fixed to obtain a self-levelling mortar, and dimensional changes, such as curling and shrinkage, were performed. Mortars with 30% OPC reached a compressive strength of 33.5 MPa and flexural strength of 7.53 MPa. A scanning electron microscope (SEM) and X-ray powder diffraction (XRD) were used to indicate the formation of N-A-S-H and a (N,C)-A-S-H gel, similar to the gel with trace of calcium. The best performance was achieved when the binary mix produced 10% OPC. A hybrid mortar of OPS-BA presented 10 times lower susceptibility to curling than an OPC mortar. The results showed that both ashes reduced the shrinkage and curling phenomena.

## 1. Introduction

Alkali-activated materials (AAM) are widely known as an eco-efficient cement due to its low CO_2_ emissions and reuse of waste residue, achieving physical and mechanical properties similar to ordinary Portland cement (OPC) [1,2,3,4]. An aluminosilicate mineral and an alkaline solution are required for activation [5]. Industrial waste, as the fly ash (FA), blast-furnace slag (BFS), and bottom ash (BA) are commonly used as aluminosilicate minerals in alkaline activation [6,7,8,9]. The development of alkali-activated systems with the use of ash from coal-fired thermal plants has proven to be promising. However, the use of BA is a potential alternative to the increasing consumption of FA as a supplementary addition to OPC clinker, or during high levels of production when materials become underused and blended into ponds as BA.

Some recent studies have demonstrated that BAs can be used as an alternative source of aluminosilicates in mixes for alkaline activation [6,7,8,9,10,11,12,13,14,15]. To ensure the sufficient reactivity of this precursor material, a larger amount of amorphous silica and relatively small particle size are required [14,15,16]. However, to develops a greater mechanical strength AAM-based depends on ash reactivity [6,11,12], and a higher rate of dissolution in alkaline medium [5]. Due to a less amorphous content, glassy film can show at the particle surface, thus hindering the initial dissolution [15]. The reactions could be improved by ashes milling [14,16] in addition to a change in the SiO_2_/Al_2_O_3_ molar ratio [17]. If the dissolution and solidification process of calcium is faster than Si/Al, then acceleration hardens [17,18,19].

Rice husk ash (RHA) is generally produced by the controlled burning of rice husks [20]. Amorphous silica is the predominant component, but its content varies according to the ash treatment [21,22]. Their use in AAM is principally an alternative source of soluble silica [23,24,25]. Kusbiantoro et al. [26] observed that the presence of RHA in alkali-activated concretes, with replacement of up to 7% of ash, increased the compressive strength and delayed the dissolution and the polycondensation of alumina, thus affecting the gelation process and increases the Si/Al molar ratio and the polymeric and the polysialate structures leading to an increase in strength.

Nonetheless, Nazari et al. [27] noticed that the binary mixtures of FA and RHA (containing 20–40% of RHA) had a larger amount of RHA reduced alumino-silicate gels phases formation. Higher NaOH molarity accelerates dissolution, althought if it accelerates above 12 M then obstruction of polycondensation occurs. The authors related better mechanical performance with higher material fineness.

Xu and Van Deventer [28] claim that Al and Si dissolve quicker when used in NaOH when compared to KOH, resulting in a more resistant structure, due to an entropy increase at the system. However, there are improvements of alkali activation when combined with activators (e.g., hydroxide and silicate). Nucleation turns slowly when used with hydroxides. When used with silicate, however, there is a higher presence of soluble silicon, which accelerates the nucleation process, acting as a “seeding” agent, rising the dissolution of vitreous phase of the ashes [29].

Palomo et al. [12] described three types of alkaline-activated cements: Cement rich in calcium, where the precursor has CaO levels >10%, in which the main products of the reaction are C-A-S-H gels; cement with low calcium content, in which the main products of the reaction are N-A-S-H gels; and the hybrid cements, with low levels of Portland cement in conjunction with the aluminosilicate, in which the products of the reaction are complex, including C-A-S-H and N-A-S-H gels.

Similarly, Garcia-Lodeiro et al. [30] established a model detailing a hybrid of 70% FA–30% OPC, activated with NaOH and a water-glass. Based on the existence of an initial dissolution of the alumino-silicate and calcium in the solution, Si and Al in alumino-silicate and of Ca-O and Si-O in the OPC broke chains. When the solution becomes saturated, a precipitation of N-A-S-H and formation of C-S-H gels occur. Over time, Si dissolves, transforming N-A-S-H chains from type 1 (Si/Al = 1) to type 2 (Si/Al = 2). In addition, the presence of Ca and Al ions in the aqueous medium that react with C-S-H gel to form a C-(A)-S-H (2D). A portion of Ca that does not react before, interacts with N-A-S-H, forming (N,C)-A-S-H gel [31].

Qu et al. [32] studied the effect of the curing temperature on hybrid cements containing FA, BFS, and 30% OPC. They found that the dominant reaction products formed were C-(A)-S-H and C-A-S-H gels. Additionally, they concluded that curing the products at 85 °C accelerated the reaction, resulting in higher mechanical strength in the initial days of curing. Yip et al. [33] reported the coexistence of C-S-H and N-A-S-H gels in hybrid cements, since the medium is not too alkaline. Furthermore, Angulo-Ramírez et al. [34] reported the presence of mainly C-S-H and C-A-S-H gels in hybrid cements with 20% OPC and 80% BFS.

There are high expectations associated with the use of hybrid cements, since they can replace the OPC cements in mortars and concretes [35]. Nevertheless, few studies have been conducted to characterize the properties of hybrid cements containing coal BA. The cure time and temperature affect the formation of the structure obtained from alkaline activation, where high reaction temperatures increase the mixes mechanical strength [36]. The potential for the application of hybrid cement to produce a self-levelling compound (SLC) was assessed in this study. For mortars comprised of SLC, the properties required are: Fluidity, low viscosity, spreading, fast drying, dimensional stability, surface resistance, and durability [37]. In other words, a self-leveling material needs to present resistance to segregation, the capacity for fluidity, and self-levelling. For the characterization of the fluidity, monopoint tests, such as mini-slump test, are widely used. Alkali-activated products usually present high viscosity, resulting from the use of an alkaline solution. This may have a positive contribution in terms of avoiding the segregation of self-levelling products. However, there are recurring problems, including high shrinkage and curling, especially when applied in materials with thin thicknesses. The shrinkage phenomena, on the other hand, is related to hardened gels of alkaline-activated systems [38].

Taking into consideration the reactivity properties of BA and RHA, the valorization of these wastes could be achieved through their use in alkali-activation. Therefore, our study produced binary and ternary hybrid systems using BA, RHA, and OPC. The samples were evaluated based upon the setting time of the fresh material, as well as their mechanical, microstructural, and durable properties. Therefore, the study sought to improve the technology of self-leveling AAM screeds.

## 2. Materials and Methods

BA, class F, was collected from the Jorge Lacerda thermoelectric plant, in South Brazil. To improve the reactivity and the formation of an amorphous phase, the ash was ground in a mill for 450 min, and treated at 600 °C for 1 h in a muffle furnace. The BA was used as the main source of aluminosilicate that produced alkali-activated mortars. The OPC used represented a cement without supplementary materials, type III according to the described on *Brazilian Standard Normalization* NBR 5733-91 High early strength Portland cement—Specification, and NBR NM 22 Portland cement with material additions pozzolan—Chemical analysis—Method arbitration [39,40]. RHA originated from a controlled combustion in a fluidized bed at the Pilecco thermoelectric plant in Alegrete, in South Brazil. The RHA was used to replace the OPC and increase the reactive silica in the ternary mixes. The chemical compounds were analyzed with an energy dispersive X-ray spectroscopy (EDX 700 Hs, Shimadzu, Tokyo, Japan). The strength activity index (pozzolanic activity) was measured according to the standard NM22 [41]. Particle size distribution was determined with laser granulometry, using a Mastersizer 2000 (Microtrac S3500, Largo, FL, USA). Fourier-transform Infrared (FTIR) spectroscopy was carried out using an Agilent Cary 600 (Agilent, Santa Clara, CA, USA) at frequencies between 4000 and 400 cm^−1^.

According to NBR 5752, the pozzolanic activity of the RHA was determined [42] as 122%. The strength activity index was the ratio of the compressive strength, with the addition and the compressive strength of control (without RHA). The strength results were greater than 75% and thus the RHA was found suitable for addition to concrete. In order to increase the surface area, the BA was ground. The grinding time was established as at least 6 h [43], until all the ground material was <45 µm. The BA was then prepared by grinding the particles in a grinding mill for 7.5 h and then put in a muffle furnace in 1 h at 600 °C for calcination. The BA calcination was performed in order to reduce the content of unburned material, due to the high ash content found in Brazilian coal.

The sodium hydroxide solution was prepared by dissolving NaOH pellets with 97% (P.A.) in distilled water, keeping a constant molar concentration of 14 for all mixes. The solution of sodium silicate (SiO_2_ = 26.5%; Na_2_O = 10.6%; H_2_O = 62.9%; and density of 1.39 g/cm³) was mixed with the hydroxide solution, keeping a proportion of 1:2 of hydroxide to silicate. Mixes of the solutions were prepared 24 h before the production of the mortar and had a final pH of 13. For the mortars production, natural fine sand dimension (1.2/0.15) mm with a fineness modulus of 1.83, and a specific gravity of 2.60 g/cm^3^ was used. The mortars were mixed in mechanical planatery mixer, with a mixing time of 2 min at 150 rpm and 1 min at 300 rpm. A superplasticizer additive, with a solids content of 0.22, based on polycarboxylate ether, was used to obtain workability (flow value > 250 mm), required for self-leveling mortars. After the mortar production, heat curing was performed for 24 h at 80 °C. Samples were then kept in a room with controlled temperature (23 ± 2 °C) and relative humidity (RH = 60 ± 5%) until the completion of the tests.

### 2.1. Preparing Alkali-Activated Mortars

We studied eight alkali-activated mortars with different mixes. In the binary mixtures, BA was partially replaced with OPC in proportions of 0%, 2.5%, 5%, 10%, and 30%. These mixes were numbered 1 (Reference), 2, 3, 4, and 5, respectively. The ternary mixtures were based on 90% BA and 10% OPC, with the OPC being partially replaced with RAH in proportions of 25%, 50%, and 75%; these were identified as mixes 6, 7, and 8, respectively. The binder and sand ratio remained constant at 1:2 (by mass) and the water/cement ratio was kept constant at 0.55 (wt %). A correction of density was necessary to equivalent volume in systems which adopt a higher volume of mineral with different specific gravity. Each mixture proportions and the molar ratio are shown in Table 1.

#### 2.1.1. Workability of the Alkali-Activated Mortars

The workability was measured using a mini slump cone (19 cm × 38.1 cm × 57.2 cm). This test was performed according to a previously described procedure [44]. The measurements were performed immediately after the mixture of the mortar, and the spreading was noted by way of four perpendicular measurements. The flow value was recorded as the average value. The maintenance of the spreading property was assessed over a period of 2 h, at intervals of 30 min.

#### 2.1.2. Mechanical Strength of Alkali-Activated Mortars

Compressive and flexural strength tests (Solotest, São Paulo, Brazil) were performed after molding the mortars in prisms with dimensions of 4 × 4 × 16 cm, according to Brazilian Standard NBR 13279 [45]. The samples were tested at 1 and 28 days after activation. An average of *n* = 3 samples to flexural tests and *n* = 6 to compressive tests. The elastic and dynamic modulus were determined according to the standard methods, NBR 8522 [46] and ASTM C597 [47], respectively. The dynamic modulus was obtained with Pundit Lab model 6.0 apparatus (Procec, Zurich, Switzerland), featuring a 20 mm diameter and 200 kHz frequency. According to the manufacturer, the accuracy of the equipment was 0.001%.

To determine the water absorption via capillarity, we used cylindrical mortars samples casted in a mold 5 cm × 10 cm dimensions, through the variation of a water column by time, in a Mariotte tube [48]. The sorptivity (absorption versus square root of time) was obtained. The wetting angles of the mixes were determined through the absorption of two different types of liquids: Deionized water and ethyl alcohol. The open porosity was determined according to the Brazilian standard NBR 9778 [49] using samples of 5 cm × 10 cm, and performing the at sample ages of 1 and 28 days.

#### 2.1.3. Resistance to Acid Attack

To assess the mortars durability, two tests were conducted: The acid attack test and the immersion and drying test. The prismatic samples with dimension of 4 cm × 4 cm × 16 cm were tested after 28 days. The acid attack was conducted by immersing the samples in 1 N solutions of hydrochloric acid (HCl) and acetic acid (HAc), separately, for four cycles of seven days. The experiment determined the mass loss, acid attack resistance, and compression strength. The wetting and drying test was also carried out 28 days after the mortars activation. The immersion and drying involved 28 cycles where each cycle consisted of keeping the samples saturated, in distilled water, for 16 h, followed by drying in an oven at 50 °C for 8 h.

#### 2.1.4. Dimensional Changes

The linear shrinkage was measured by drying according to NBR 15261 [50]. Samples with dimensions of 2.5 cm × 2.5 cm × 28.5 cm were used to measure the linear variation. The curling phenomenon was evaluated by measuring the vertical displacement of a mortar sample placed in a 33 cm × 33 cm × 3 cm mold, using four LVDT (Linear variable differential transformers) sensors at the mortar screeds, at distances of 2.5 and 4.5 cm from the edges, and two sensors in the center of the mortars [51]. Mortars were mixed in portable vertical shaft mixer. Lastly, mass loss was monitored with a loading cell placed under the screeds.

### 2.2. Preparing Alkali-Activated Pastes

A selective chemical attack of alkali-activated pastes was performed after 28 days of heat treatment (24 h at 80 °C). Due to the ash dissolution in the alkaline activation, a concentration of 1:20 HCl was used to determine the quantity of reacted cement, thus evaluating the degree of reaction [6]. The 1:20 HCl solution was prepared using HCl with 37% of distillated deionized water.

Besides that, we investigated setting time and microstructure. X-ray diffractograms of the samples in powder form were obtained using a Phillips X-pert CuKa diffractometer (Philipps Analyticlal XRay, Almelo, The Netherlands, 40 kV, 30 mA), with scan step size of 2° s^−1^, at 2θ angle range in 3–55° divergence slit of 1° and receiving slit of 0.2-mm. For the scanning electron microscope (SEM) analysis, sample particles were observed under a Jeol JSM-6390SL (JEOL USA Inc., Peabbody MA, USA) (15.0 kV) and energy-dispersive X-ray spectroscopy (EDS) probe was also performed at the Central Laboratory of Electron Microscopy (LCME-UFSC). A fourier Transform Infrared spectroscopy (FTIR) was performed for characterize microstructure, using a JASCO FT-IR 4200 (JASCO Coorporation, Tokyo, Japan) at frequencies between 400 and 4000 cm^−1^. The measurements were performed with KBr pellets. For the pastes the proportions of cement material and alkaline activator (ratio binder:solution) and superplasticizer were kept the same. The setting time was determined using a Vicat apparatus (Solotest, São Paulo, Brazil) applying the same cure conditions used for mortar casting.

### 2.3. Microstructure Analysis

All mortar samples were subjected to microstructural analysis after 28 days, in order to verify the effect of the proportion of OPC in the BA replacement, as well as the influence of reactive silica. Powdered material (<150 µm) was analyzed with X-ray powder diffraction (X-pert system Philips Analytical XRay, Almelo, The Netherlands). Fragments were used to obtain SEM images (JEOL USA Inc., Peabbody, MA, USA), with the same devices used to the pastes investigation.

**Figure 1:**
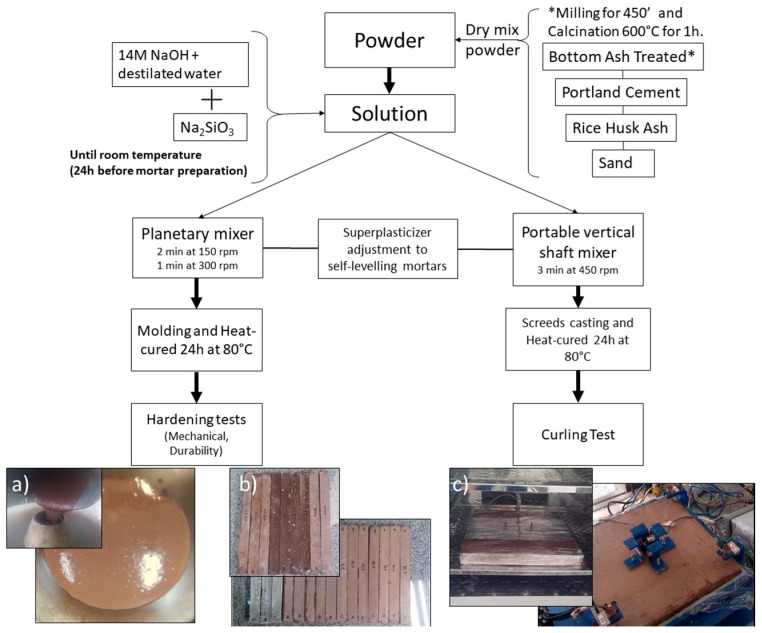
Experimental procedure schema: (**a**) mini-slump flow and self-levelling mortar (spread); (**b**) linear shrinkage bar tests with binary samples (up) and ternary samples (down); (**c**) heat cure of screeds of and curling test.

Below, Figure 1 summarizes our experimental procedure.

## 3. Results

### 3.1. Precursors Characteristics

Below, Table 2 summarizes the results obtained for the chemical and physical characteristics of the precursor minerals. 

**Figure 2:**
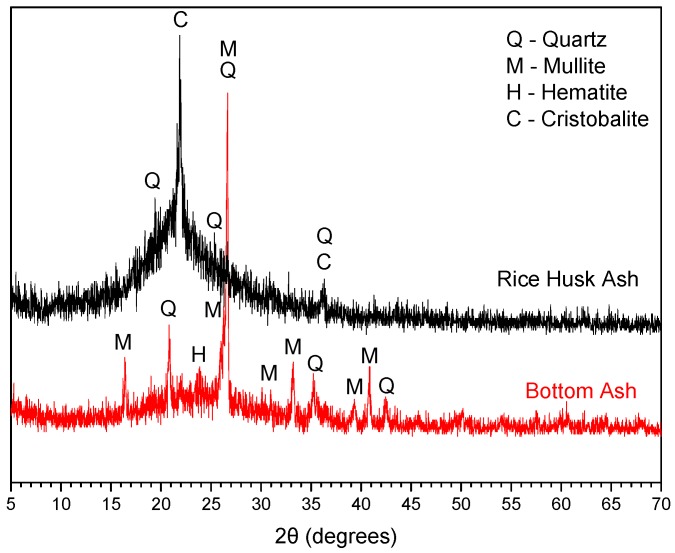
XRD patterns of bottom ash and rice husk ash.

**Figure 3:**
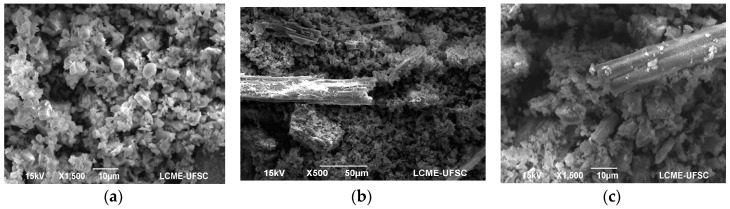
Singular electron microscope (SEM) images: (**a**) Bottom ash after grinding and calcination at 600 °C (×1500); (**b**) Rice husk ash (RHA) (×500); (**c**) RHA (×1500).

The BA and RHA contained >40% reactive SiO_2_ and the content of vitreous phase was >50% [6,7]. The average particle sizes were below the diameter of OPC—8.59 µm and 10.49 µm for BA and RHA, respectively. The specific areas of the RHA and BA ashes were compared with OPC (500.5 m^2^/kg), with values of 1174 and 715.5 (m^2^/kg), respectively. Figure 2 shows the X-ray diffractogram of BA after treatment and the RHA. RHA ash contains mainly amorphous materials, with crystalline phases of cristobalite (PDF#39-1425) and quartz (PDF#46-1045). The RHA particles had an irregular shape and were lamellar and fibrous, with presence of elongated particles, due to the unburned material, with lengths of around 150 µm (Figure 3). BA mainly contains quartz crystals, mullite, and hematite, with mostly spherical particles and unburnt carbon (loss on ignition (LOI) = 6.67%).

The grinding of the BA particles promoted a reduction in the internal porosity, the formation of irregular particles, and the presence of microspheres (Figure 3a). The RHA particles exhibit irregular, lamellar, and fibrous materials related to the presence of unburned material (Figure 3b).

**Figure 4:**
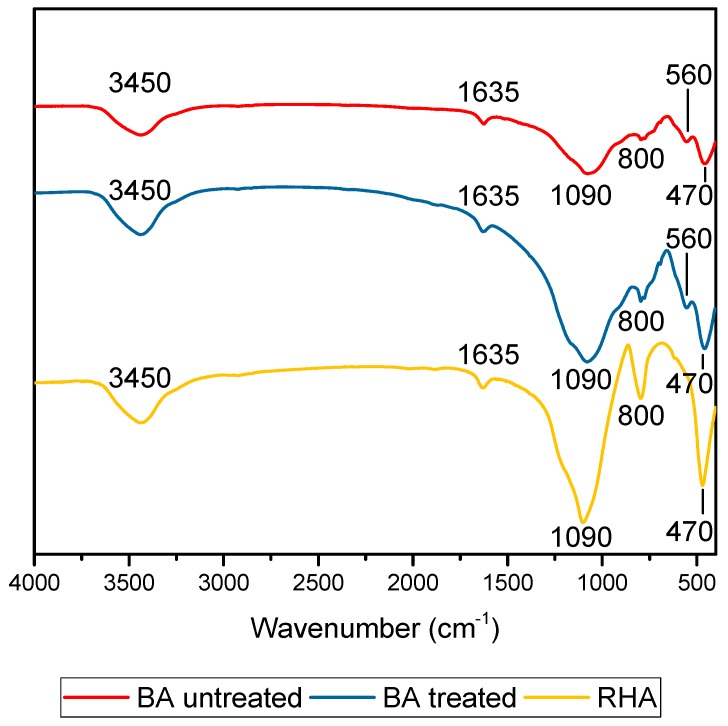
Fourier-transform Infrared (FTIR) spectra for precursor minerals: Bottom ash (BA) untreated, BA treated, after grinding and calcination at 600 °C (1 h); Rice husk ash (RHA), from commercial supplier.

FTIR spectroscopy was facilitated using an Agilent Cary 600 instrument at frequencies between 4000 and 400 cm^−1^. Figure 4 shows the FTIR results for the minerals (treated and untreated BA and RHA). The wide band located at 3600 to 3200 cm^−1^ for both samples was due to the O-H bond. The band close to 1600 cm^−1^ corresponded to the angular deformation of the H-O-H bond in the raw materials. The band present at 1200 and 800 cm^−1^ was due to the stretching vibration of amorphous Si-O-Si, which showed a higher content to RHA due to its transmittance. The small band close to 500 cm^−1^ for BA was due to crystalline Si-O-Si. The band at 560–550 cm^−1^ can be associated with the octahedron present in the mullite. The BA heat treatment at 600 °C increased the intensity of the O-H band and the peak at 1551 cm^−1^ appeared because the strongest vibration was the C-O bond.

### 3.2. Workability of the Alkali-Activated Mortars

We obtained hybrid alkali-activation mortars with self-leveling properties. All mixtures were tested with a superplasticizer based on polycarboxylate, except for OPC 10, which was also prepared with an additive based on naphthalene. There was a trend toward greater flowability for mixes with lower OPC content. The self-leveling properties were acquired with 1.0–1.4% of superplasticizer. OPC 5 required the same superplasticizer content as OPC 30 sample, due to their lower workability. Self-leveling properties were also obtained for all ternary mixes by adding 1.2% of additive (by mass) to the binder. No exudation or segregation was observed in the final mini-slump test.

Mortar samples that reached an initial flow value were 250 mm. After 30 min, the binary mix spreads were: 280, 225, and 225 for OPC 2.5, OPC 5 and OPC 10, respectively. For ternary mixes, the spreads after 30 min were 225 ± 25 mm. Reductions of more than 60%, compared to initial flow value, occurred after 60 min.

The final setting after heat alkali-activation were 18 to 20 min, a rapid hardening to self-leveling product.

### 3.3. Mechanical Strength and Durability of the Alkali-Activated Mortar

Table 3 summarizes the strength values and physical parameters (sorptivity, porosity, and wet angle) found at day 1 and day 28.

The development of compressive strength for the different amounts of added OPC indicate that the mean values did not differ significantly. Low strength was noted for the samples containing only BA, due to the low degree of reactivity observed in the selective acid attack on the pastes. For OPC 0 and OPC 2.5, there was no significant difference in the strength for later ages. When comparing the average values of OPC 2.5 and OPC 5, no significant difference after one day of curing was revealed. Therefore, it was verified that higher levels of cement influenced the development of the compressive strength. The flexural strength results for the binary samples demonstrated similar behavior, with values five times lower than those with the compressive strength. There was a delay to the reaction of bottom ashes (OPC 0).

When RHA was used as replacement material in the mix OPC 10 in proportions of 0%, 25%, 50% and 75%, there was a reduction in the strength [12]. The elongated RHA particles indicate a lower degree of ash dissolution. The reduction in the amount of CaO present also decreased the reactivity. Higher compressive strength values can be observed with lower amounts of RHA, indicating that RHA had low reactivity. The flexural strength was lower than that of the binaries mixes, due to the degree of reaction.

It was found that at the binary mixes dynamic modulus at 28 days, higher values were obtained by increasing OPC content in the mixes; for ternary mixes, the values decreased with higher RHA content. Dynamic modulus of elasticity was 10.5 to 18.5 GPa.

For all mixes, the initial sorptivity after day 1 was higher than that observed after 28 days. With an increase in the OPC content in the binary mixes after alkaline-activation, an increase in the absorption can be observed after day 1 of aging, which was associated with increased porosity. After 28 days, we observed that for the lower levels of BA replacement in OPC (OPC 2.5 and OPC 5 samples) there was higher sorptivity when compared with OPC 10 and OPC 30. This was due to the formation of (N,C)-A-S-H gels in the activations when a higher OPC content was present, thus resulting in the filling of the pores.

In addition, we observed that all ternary mixes exhibited higher sorptivity when compared with the reference sample. Similar behavior was evident after 1 and 28 days with a reduction in the sorptivity. RHA 75 showed low sorptivity, primarily due to the inert nature of the RHA, which showed lower reactivity and filled the capillary pores.

Every sample had a higher open porosity after day 1, compared with the result after 28 days. An increase in the OPC contents resulted in lower open porosity and absorption, due to an increase in the (N,C)-A-S-H gel formation. In addition, the ternary mixes showed lower open porosity and absorption, which we attributed to the filler effect due to presence of RHA.

#### Durability

**Figure 5:**
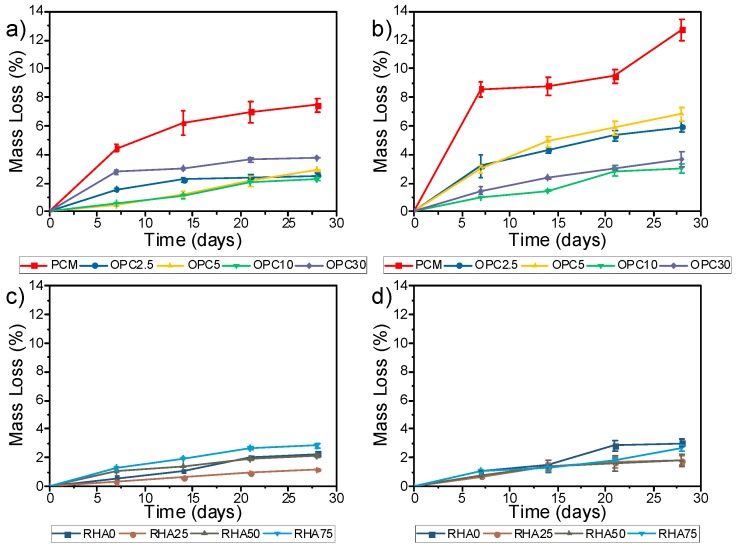
Mass loss of binary and mortars during acid attack: (**a**) Binary after HAc exposure; (**b**) Binary after HCl exposure; (**c**) Ternary after HAc exposure; (**d**) Ternary after HCl exposure.

Figure 5 shows that the mass loss was higher for the binary mixes compared with the ternary. This is due to the attack on the OPC, the (N,C)-A-S-H gel, and the alkaline activation BA gel (N-A-S-H). It can be noted that the mixes containing 2.5% and 5% of OPC showed a significant degree of degradation, indicating that OPC content affected the resistance of the acid attack. In the mortars containing RHA, the degree of degradation was lower. Comparatively, the results show that the Portland cement mortar (PCM), with the same *w*/*c* ratio, degraded more aggressively than the AAMs. For the AAM, no signs of surface deterioration were found in the visual inspection of the prisms. The OPC specimens were severely deteriorated after the 28 cycles.

**Figure 6:**
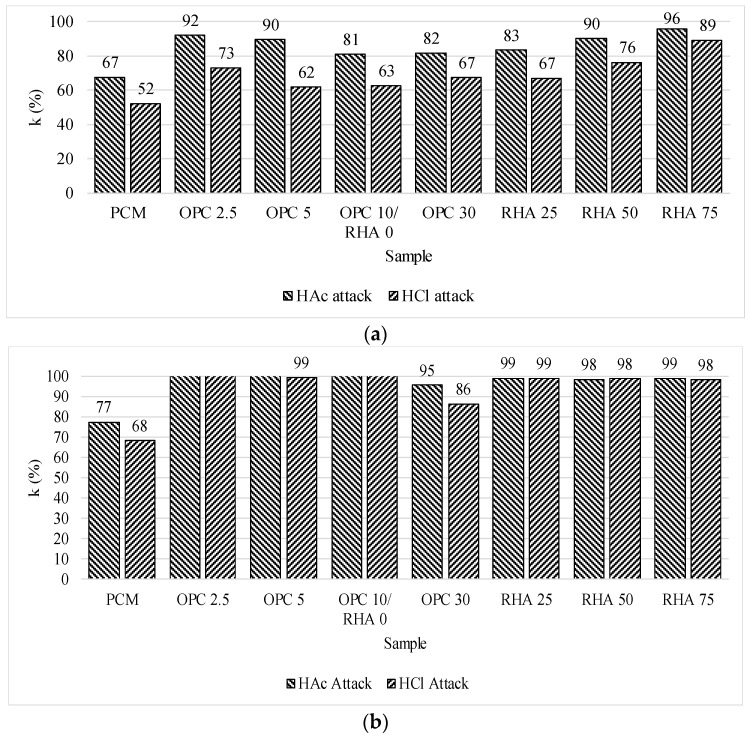
Resistance to acid attack: (**a**) Flexural strength; (**b**) Compressive strength. K value (%): K = ((Resistance at 28 days − Resistance after cycle)/Resistance at 28 days) × 100.

Figure 6 shows the results after the HAc and HCl exposure. The flexural strength was strongly affected by the acid attack, due to the formation of cracks in the mortar, as shown in Figure 6a. However, for all alkaline-activated samples, no significative compressive strength loss was observed (Figure 6b).

The results of the wetting and drying tests carried out on the mortars are shown in Table 4. It can be observed that 28 cycles of wetting and drying did not cause degradation of the PCM samples. The alkali-activation had an influence on the flexural strength, mainly in the case of binary mixes. Only the alkali-activated sample containing the highest percentage of Portland cement showed lower results after wetting and drying cycles.

### 3.4. Dimensional Changes

#### 3.4.1. Linear Shrinkage

**Figure 7:**
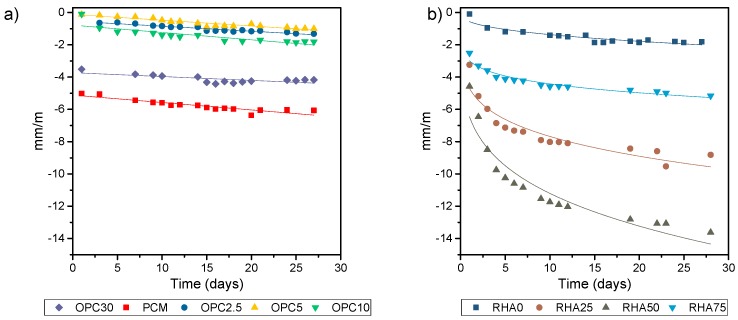
Drying shrinkage: (**a**) Binary mortars; (**b**) Ternary mortars.

The results for linear shrinkage variation are shown in in Figure 7. We found that binary samples had a lower linear shrinkage than ternary samples. In binary mixes, high contents of OPC raised the linear shrinkage, due to the amount of Portland paste, which increased gel formations. RHA was increased in three times more mortar shrinkages, due to a higher presence of unreacted NaOH, a which facilitated the carbonation of these samples.

#### 3.4.2. Curling Effect

The curling effect for binary mixes was performed for all mortars screeds with a mechanical strength value of >20 MPa (at day 1).

**Figure 8:**
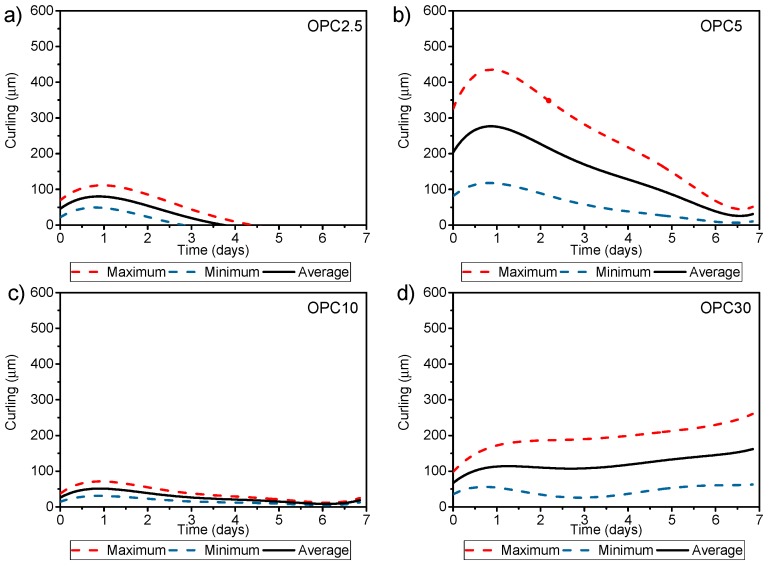
Curling over the time for binary mortars screeds. (**a**) OPC 2.5 mortar mix; (**b**) OPC 5.0 mortar mix; (**c**) OPC 10 mortar mix; (**d**) OPC 30 mortar mix.

**Figure 9:**
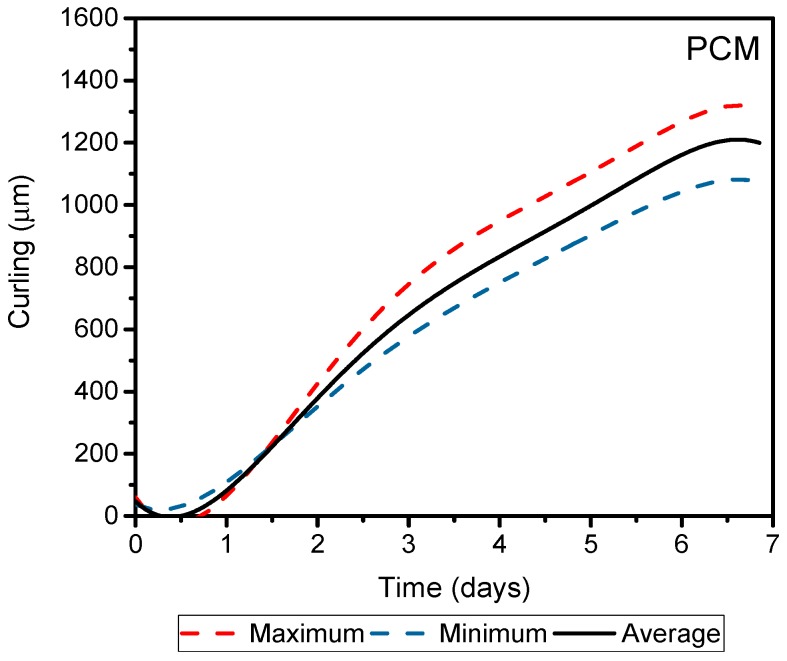
Curling over the time for Portland cement mortars screeds.

Figure 8 shows screeds warping in binary mortars. Figure 9 shows mortars comprised only of Portland cement mortar (PCM), with same *w*/*c* ratio as alkali-activation (0.55). In addition, the maximum, minimum, and average value of each analyzed warpage is shown in Figure 9.

We observed a difference in the curling of mortars when comparing cement compositions, specifically between mixes with a 10% of cement compositions and OPC. A maximum warpage obtained for these samples occurred on the first day, bearing in mind that the temperature gradient was high inside the screeds until it reached the surface. Following this rapid water evaporation, probable micro internal cracks appeared in these alkaline-activated mortars with low cement content. These micro cracks served as warping compensators, resulting in the reduction of the lifting of the edges. Mortar screeds that contained 30% of OPC demonstrated a similar behavior to mortar screeds with only Portland cement. The initial behavior of this mortar was the same as described for the other mortars of alkaline activation, presenting an early lifting of edges on the first day of testing. The maximum average warpages of alkaline activation screeds represented only 7%, 25%, 5%, and 14% for OPC 2.5, OPC 5, OPC 10, and OPC 30, respectively, of the average warpage obtained for the mortar PCM. This revealed a 10 times reduction of warpage when used alkaline activation mortars.

**Figure 10:**
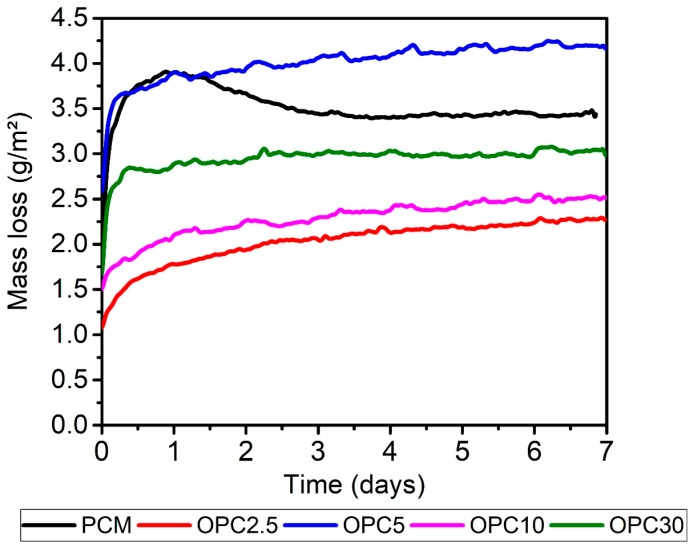
Mass loss during curling testing of mortars screeds: Binary mortars and Portland cement.

The OPC 5 screeds had the highest average warpage among the results. This was attributed to the fact that this mixture required greater use of superplasticizer (1.4% of the mass of the cement material). The mass variation related to surface during the test can be seen in Figure 10.

The sample OPC 5 had the highest curling due to mass loss; OPC 30 had the second highest mass loss. However, there was an inversion between the samples OPC 2.5 and OPC 10. Except for the sample OPC 5, all the alkaline activations obtained lower mass loss when compared to PCM. Higher mass loss was observed at hybrid mortars when compared with PCM; otherwise, hybrid mortars showed lower shrinkage and curling rates once the water in PCM was present between C-S-H layers. Water in alkali activation was not chemically incorporated in N-A-S-H or (N,C)-A-S-H gels, and acted as free water in the system [22]. Another factor that determined water results was polymerization, which was observed using FTRI bands.

**Figure 11:**
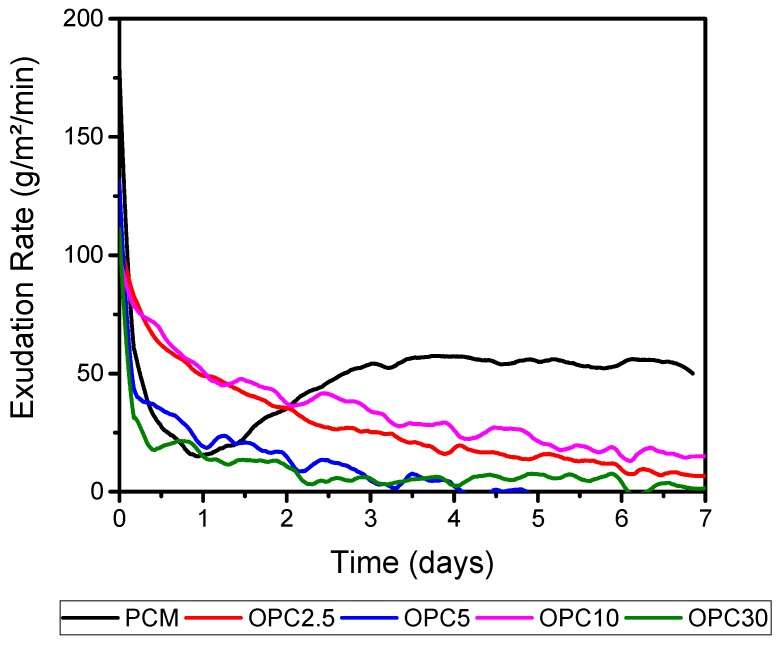
Exudation rate during the curling of mortar screeds.

Figure 11 shows the exudation rate in g/m²/min of the mortars. This calculation was performed using the ratio of mass, which was obtained through its variation using the superficial area of the screeds (33 cm × 33 cm). Similar behavior was observed within the variation of mixtures masses, displaying higher rates for the mortar of OPC 5, OPC 30, OPC 10, and OPC 2.5 respectively. PCM presented a higher exudation rate.

### 3.5. Microstructural Analysis

#### Alkaline-Activated Pastes

After 28 days of curing, an attack with an acid solution of 1:20 HCl was performed to the pastes, which were ground to less than 45 µm. The attack was used to dissolve the reaction products formed through the alkaline activation process. This parameter assumed the dissolution of reacted phases of BA and OPC. Through the selective chemical attack, we obtained the reaction degree of each mixture (Table 5). The lowest reactivity (σ = 25.49) was observed for mixes containing only BA as a mineral, since its reactions processes, as well as its responses of strength, were very low. The OPC presence generated products with a higher reaction degree, attributed to the rapid dissolution and larger formations of gel (N,C)-A-S-H. Mixes with OPC also demonstrated a faster dissolution and gel polymerization. This effect was mainly identified in samples containing more than 10% of OPC and a reaction degree greater than 50. The SEM images, setting time, and strengths reinforced this finding. In contrast to ternary mixtures, the presence of RHA inhibited the reaction degree, showing all reactions less than 50. This finding suggest two hypotheses when considering the influence of ternary mixtures: the less OPC content, the less (N,C)-A-S-H gels; RHA increased in an Si gel formation. Thus, it was concluded that the OPC contributed to the formation of activated gels.

**Figure 12:**
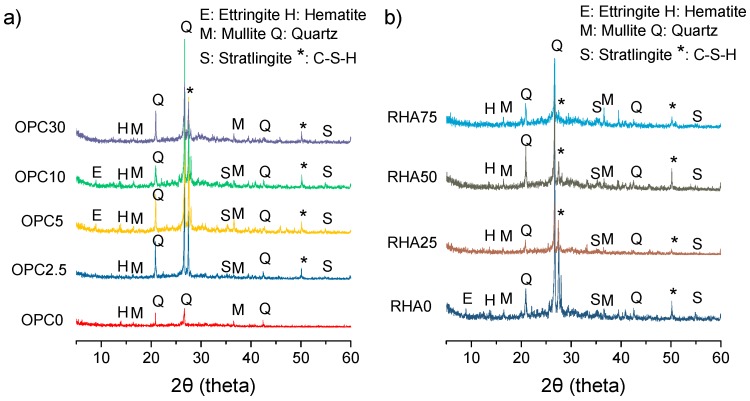
X-ray diffractogram patterns: (**a**) Binary mixes (**b**) Ternary mixes samples.

Figure 12 shows the X-ray diffractogram patterns for the alkali-activated samples (at 28-days), which reveal the presence of quartz crystals (PDF# 46-1045) and mullite (PDF# 15-0776), due to the unreacted material. The presence of a significant amorphous content can be noted by the halo formed between 2θ at 25–30°, which increased as the contents of OPC increased. Ettringite (PDF# 41-1451), Stratlingite (PDF# 29-0285), and C-S-H (PDF# 42-538) peaks were observed for samples with over 10% of OPC, due the normal hydration of OPC. No C-A-S-H formation was observed in binary mixtures. This finding was not in agreement with results reported in the literature [9]. Other findings concluded that high calcium content and pH levels favored the formation of C-A-S-H rather than N-A-S-H or (N,C)-A-S-H gels. But the sample without the Portland cement (OPC 0 Figure 12a) presented lower peaks (close to the base line), thus verifying its low reactivity. It can be observed that the same crystalline phase present on BA or RHA remained in alkali-activated samples (at 28 days).

It can be observed that the same crystalline phase found in BA and RHA were present in alkali-activated samples (at 28 days).

**Figure 13:**
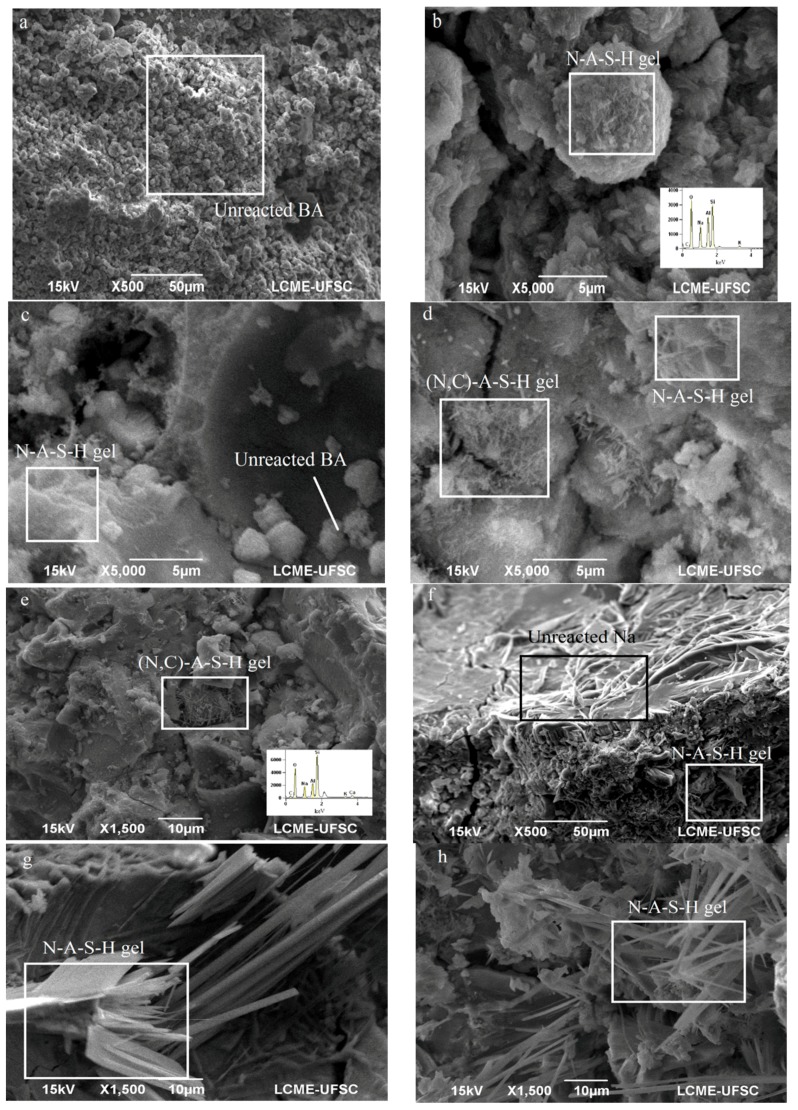
SEM analysis samples at 28 days: (**a**) OPC 0; (**b**) OPC 2.5 with EDS microanalysis from N-A-S-H gel; (**c**) OPC 5; (**d**) OPC 10; (**e**) OPC 30 with EDS microanalysis from (N,C)-A-S-H gels; (**f**) RHA 25; (**g**) RHA 50; (**h**) RHA 75 at 28 days.

The SEM images presented in Figure 13 show sample OPC 0, a high content of unreacted BA. Because of its lack of silicate presence, there was no N-A-S-H gels and no (N,C)-A-S-H gels, in large part due to the absence of CaO. Figure 13b shows OPC 2.5 and its presence of N-A-S-H gels with no traces of calcium phases. Figure 13c displays OPC 5, as well as particles of unreacted BA. Figure 13d presents OPC 10, which had traces of calcium in geopolymer gels, the forming gel (N,C)-A-S-H. Figure 13e presents the same gel with calcium, for OPC 30. Figure 13f shows RHA 25 with unreacted sodium and the gel N-A-S-H. Figure 13g and h show RHA 50 and RHA 75, respectively, both featuring long crystals of gel N-A-S-H.

**Figure 14:**
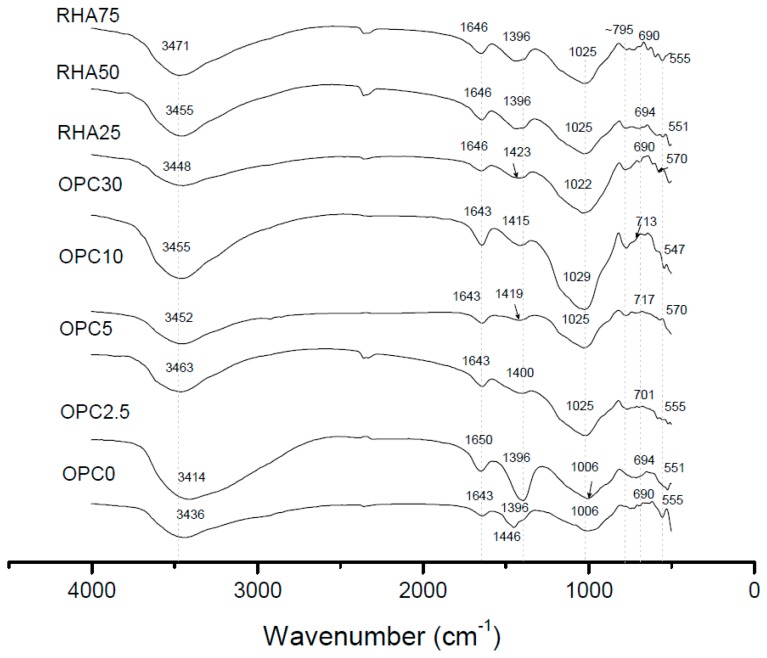
FTIR of the binary and ternary samples at 28 days of age.

Figure 14 shows the 28 days FTIR spectra for binary and ternary mixtures. The bending vibration (547–570 cm^−1^) corresponded to the Si-O-Al bonds. The wavenumber 690–717 cm^−1^ was attributed to Si-O symmetrically stretching vibrations [51]. The spectrum band at ~795 cm^−1^ was attributed to quartz, except for sample OPC 0 [52,53]. The vibration corresponding to N-A-S-H gel band (T-O, where T = Si or Al) was seen at 1006–1029 cm^−1^ [51]. Higher Portland cement content increased the band intensity, indicating an increase in gel formation. Also, the increment of OPC content shifted the band to a higher wavenumber, which we attributed to a replacement increase of AlO_4_ to SiO_4_ tetrahedron. For ternary mixes, an increased RHA content reduced the intensity of N-A-S-H gels.

Carbonates (1396–1423 cm^−1^) bands υ_3_ corresponding to Na_2_CO_3_ [53] were mainly observed in lower Portland cement contents. For OPC 0, we observed a second band at 1446 cm^−1^ that also corresponded to carbonated ions (CO_3_^2−^). This behavior agreed with the reaction degree, due to a minor soluble calcium upon the alkali activation. This resulted in more unreacted sodium content that reacted with the atmosphere. Hanjitsuwan et al. [17] concluded that higher strengths are achieved with reduction in carbonate and O-H bands. Bands at 1643 cm^−1^ corresponded to bending vibrations and ~3450 cm^−1^ to tensions of O-H bond in water. The FTIR spectra analysis reinforced the results obtained in the compressive and flexural strength and microstructural analysis.

## 4. Discussion

A self-leveling hybrid mortar consisting solely of Portland cement achieved the best mechanical strength (above 20 MPa on day 1) and durability. SEM analyses showed that hybrid mortar with more than 10% of Portland cement produced gels with traces of calcium, which increased the durability and strength of mortars. For mixes containing <10% OPC, only traces of N-A-S-H gels were observed. The RHA used to obtain the ternary mixes contained inert particles, due to unburned material, contributing to a decrease in gel formation.

The alkali activation containing BA proved unsatisfactory, due to a lower reaction degree and strength. To reach a good performance, the BA was ground (450’) and underwent calcination at 600 °C, thus increasing the Si/Al with sodium silicate, a thermal cure at 80 °C, and sodium hydroxide to molarity (14 M). According to prior research [11,12,13,14,15,16], there are several key properties influencing the reactivity of industrial ashes with low calcium content, including particle size (<45 μm), highly reactive silica content, and vitreous phase content with a lower unburned percentage.

In this study, we observed the coexistence between Portland cement hydrates (C-S-H, ettringite, and stratlingite) and the alkali-activated product (N-A-S-H gel). This fact contributes to a better performance of mechanical strength and durability. Studies shows that alkaline with a medium temperature accelerated the reaction of Portland cement phases [34]. This contributes to an improvement of strength with higher Portland cement content to hybrid mortars. The sulphate presence developed the ettringite and stratlingite phases, however, no U phase (sodium-substituted AFm phase) was observed at 28 days, due to the reaction of sulphate that formed ettringite and stratlingite. Within ternary mortars, we observed a reduction of C-S-H and N-A-S-H gel formations. The SEM analysis showed a N-A-S-H crystalline phase as an elongated needle, which increased the porosity and presence of unreacted sodium, showing a poor reaction degree for the samples. Our hypothesis was that the silica derived from RHA contributed to the crystallization N-A-S-H gels, forming a zeolite crystal (Figure 13g,h). However, the zeolite detection using XRD or FTIR occurred after 28-days of cure [50]. Furthermore, we observed a formation of silicate crystals, provided by the increase of silica soluble.

With reference to sorptivity and open porosity, we observed an increase of OPC content, due to an elevated formation of (N,C)-A-S-H and N-A-S-H, causing a reduction of the capillary pores. However, with the increase of rice husk ashes in ternary mortars lower sorptivity occurred. The decrease of CaO content reduced its reactivity, but the inert nature of the RHA still filled the capillary pores. Other authors have observed similar microstructures with lower contents of RHA or nano-silica [26,45]. The open porosity of all samples was higher at day 1 compared with the result at 28 days, which was in part explained by the ongoing formation of the gels. An increase in the OPC contents resulted in lower open porosity and absorption, due to an increase in the (N,C)-A-S-H gel formations. In addition, the ternary mixes showed lower open porosity and absorption, which we attributed to the filler effect [45].

The drying shrinkage of hybrid mortars was affected by the calcium content from OPC. All the binary mortars showed a lower drying shrinkage when compared to PCM. Heat hardening created a denser matrix that increased rigidity of the solid network, improving the resistance to the drying shrinkage [38].

In binary mortars, the increase of calcium content exhibited a higher drying shrinkage. The most important drying shrinkage occurred until day 30; after this the shrinkage decreased at a slow increment. These results were associated with the increased initial porosity of higher calcium content, as seen with the 1-day sorptivity data. The main factor was the delay of C-S-H formation. This occurred due a lower solubility of Ca^2+^ from OPC after an increase in pH [45]. Until the ultimate formation of NASH gels, the presence of OH^−^ is reduced and the C-S-H formation begins. The pore filled with higher OPC content was observed during 28-day sorptivity. This explains the higher initial drying shrinkage, followed by the larger mechanical properties (compressive strength, elastic modulus).

The drying shrinkage was higher to specimens with higher silica and sodium content [38]. Therefore, linear shrinkage was greater to ternary mortars due to a higher presence of unreacted NaOH (seen in Figure 13), facilitating the carbonation of these samples and resulting in higher values of shrinkage. Other factors were that higher shrinkage had a lower reaction degree, due to its slow microstructure development and low mechanical properties. The possible zeolite formation also contributed to higher porosity and higher drying shrinkage, seen with its high sorptivity and its low decrease from 1 to 28-days (Table 3).

The curling analysis showed that at the first day occurs an accelerated water evaporation, main affecting the AAM with low OPC content (up to 10%). This is attributed to the micro internal cracks that the evaporation caused. These micro cracks served as warping compensators, resulting in the reduction of the lifting of the edges. OPC 30 expressed similar behavior to PCM. However, the curling effect of the hybrid cement was 10 times lower than the PCM. OPC 5 showed the highest curling and mass loss to the mortar screeds, due to the effect of its higher content of the superplasticizer additive (1.4%). According to the rate of exudation, the hybrid mortar showed a higher rate until day 2; after that it expressed a lower rate of exudation, proving its reduced drying shrinkage. The larger proportion of the pore capillary from OPC 5 showed no evolution of time, presenting greater sorptivity after 28-days. These results explain the mass loss when emptying water through capillary pores [38].

Most of OPCs durability problems were associated with the dissolution of its phases. OPC was rich in calcium, which precipitated in expansion products as gypsum or ettringite [52]. Nevertheless, the low Ca content in AAM acted as an advantage for the acid attack. During the wetting and drying tests, we observed a higher content of OPC, which reduced the mechanical strength and only affected the OPC 30 sample. This was due to two possible hypotheses: (1) A greater deterioration of calcium disillusion in (N,C)-A-S-H gel structures [52] or (2) a calcium-free mortar during the procedure of wetting and drying. However, visual variation was not observed after 28 cycles of wetting and drying.

All the hybrid mixtures showed a higher resistance to acid when compared with mortar consisting of only Portland cement. That happened because the absence of aluminum in the main reaction formed. The presence of aluminum within Na^+^ ions increased the acid character of gel, improving stability in an acid medium C-S-H gel, derived from OPC hydrated with water [52]. A second hypothesis was that the acid attack decalcifies the AAM, resulting in the formation of calcium acetate and an aluminosilicate gel layer that prevents acid penetration [52,53]. This fact indicated that higher calcium content resulted in lower mass loss, as observed in Figure 10.

All the mortars showed good wettability (wetting angle lower than 90°), presenting a hydrophilic surface, a propriety indicated for further adhesion of mortars to substrates. It was also verified that all of the hybrid alkaline activations had 10 times less shrinkage than mortars from Portland cement. OPC 10 was the best mortar choice, due to the increased presence of reaction products, such as (N,C)-A-S-H. This resulted in a better compressive strength, sorptivity, wetting angle, resistance to acid, and drying shrinkage close to the hybrid mortars, with a low content of cement. OPC 30 had quick flowability loss, stiffing, and hindering self-leveling properties.

Despite the response of selective chemical attack (1:20 HCl), the dissolution of products may have also affected some mineral phases present in the ashes.

According to these results, we conclude that it is possible to perform alkali activations, with low amounts of Portland cement, 2.5% mass of binder materials, durable materials, and the inclusion of materials with similar behaviors to those of mortars of Portland cement. It was possible to carry out self-levelling mortars of alkaline hybrid activation, with valorization of bottom ash. When used as ternary source RHA reduced mechanical strength and dimensional variations were more important, and not achieving a strength necessary to a self-levelling mortar screed at day 1.

## 5. Conclusions

An alkali-activated material with high content of low-quality BA (70% to 97.5%) can be mixed with a smaller amount of Portland cement. This blended cement presented great mechanical strength, even with 2.5% OPC, achieving for 1 and 28 days of curing, 11 and 8.4 times higher compressive strength than the reference (0% OPC), respectively. For hybrid cements of 70/30 (BA/OPC), the compressive strength was 14.5 and 13 times higher in the 28 days of curing. Even with very low content, the presence of calcium provided from OPC was the key to achieve mortars with good mechanical and durability properties. The main conclusions are:

- Coexistence of alkali activated gel and Portland hydrates (C-S-H gel and ettringite) is responsible for the increased mechanical properties for hybrid mortars, resulting in a denser matrix;
- An increase of OPC content accelerated the pore filling at later ages occurs, and the shrinkage and curling effects of binary mortars are higher and depending on the reactivity calcium content;
- The silica from RHA improve the crystalline zeolite formation, resulting in a more durable mortar but with a decrease of strength. The conversion of N-A-S-H gels from zeolite increase the porosity due formation of elongated needles;
- The ettringite formation contributes to a better performance of immersion-drying tests to higher calcium content. OPC 30 did not form ettringite and it was the cause of sample degradation;
- The beneficial effect of hybrid mortars to reduced liner shrinkage (up to 84%) and curling effect in screeds (10 times lower) compared with Portland mortars conventional show the great behavior to use these binary mixtures as self-leveling mortars;
- The alkali activation OPC 10 showed best results set with good workability properties, compressive strength (28.21 MPa), dynamic modulus (16.05 GPa), lowest sorptivity (0.0084 cm√min), lower curling screed (<100 μm) and the lowest mass loss to acid attack.

## Figures and Tables

**Table 1:** Mixture proportions.

Mix	Samples	Mortar Mix Proportions (per 100 g of Precursor)							Molar Ratio		
		BA	OPC	RHA	SH ^1^	SS ^2^	Sand	SP ^3^	Si/Al	Na_2_O/SiO_2_	H_2_O/Na_2_O
1	OPC 0 (REF)	100	-	-	60	30	200	1.0	2.57	0.27	12.26
2	OPC 2.5	97.5	2.5	-	60	30	200	1.0	2.60	0.27	
3	OPC 5	95.0	5.0	-	60	30	200	1.4	2.64	0.27	
4	OPC 10/RHA 0	90.0	10.0	-	60	30	200	1.2	2.71	0.28	
5	OPC 30	70.0	30.0	-	60	30	200	1.4	3.08	0.30	
6	RHA 25	90.0	7.5	2.5	60	30	200	1.2	2.81	0.27	
7	RHA 50	90.0	5.0	5.0	60	30	200	1.2	2.91	0.26	
8	RHA 75	90.0	2.5	7.5	60	30	200	1.2	3.02	0.25	

^1^ Sodium hydroxide solution. ^2^ Sodium silicate solution. ^3^ Superplasticizer.

**Table 2:** Chemical and physical characteristics of the precursor materials.

**Total Oxide (wt %)**	**SiO_2_**	**Al_2_O_3_**	**Fe_2_O_3_**	**CaO**	**K_2_O**	**SO_3_**	**TiO_2_**	**LOI ^1^**		
BA	40.82	37.46	5.71	1.73	5.20	0.29	1.90	6.67		
RHA	92.00	ND ^2^	0.17	0.60	1.72	ND ^2^	0.11	3.50		
**Particle Size**										
	**D_10_ (µm)**		**D_50_ (µm)**		**D_90_ (µm)**		**Specific Gravity (g/cm^3^)**		**Blaine Fineness (m^2^/kg)**	
BA	1.545		8.597		22.733		2.43		715.5	
OPC	3.561		15.822		60.120		3.05		500.5	
RHA	2.166		10.497		41.208		2.12		1174.0	

^1^ Loss on ignition; ^2^ ND: Not detected.

**Table 3:** Strengths values and physical parameters at 1 and 28 days for mixes of ordinary Portland cement (OPC) and RHA.

Mixes	Compressive Strengh (MPa)		Flexural Strengh (MPa)		Sorptiviy (cm√min)		Wet Angle (°)		Open Porosity		E (GPa)
	Day 1	28 Days	Day 1	28 Days	Day 1	28 Days	Day 1	28 Days	Day 1	28 Days	28 Days
OPC 0	1.92 (1.12)	2.58 (0.13)	1.35 (0.20)	1.60 (0.22)	0.0158	0.0159	89.76	89.76	25.30	22.74	---
OPC 2.5	21.27 (1.64)	21.59 (2.40)	4.61 (0.12)	7.38 (0.29)	0.0205	0.021	80.84	80.84	20.05	15.22	14.68
OPC 5	22.34 (1.31)	23.22 (2.40)	3.89 (0.18)	7.98 (0.20)	0.0361	0.031	76.04	76.04	22.91	14.08	14.78
OPC 10	26.01 (1.69)	28.13 (2.21)	4.86 (0.29)	7.75 (0.49)	0.0409	0.0084	88.65	88.65	15.80	15.62	16.05
OPC 30	28.00 (1.10)	33.46 (2.08)	4.67 (0.49)	7.53 (0.23)	0.0445	0.0215	85.28	85.28	15.89	12.17	18.73
RHA 25	11.46 (0.68)	20.14 (2.11)	3.47 (0.21)	4.05 (0.29)	0.0363	0.0306	89.95	89.92	12.21	10.9	13.91
RHA 50	6.13 (0.25)	14.30 (1.52)	2.56 (0.12)	3.60 (0.11)	0.0471	0.0197	89.91	89.97	13.20	12.22	12.62
RHA 75	5.76 (0.24)	12.74 (0.62)	2.48 (0.13)	3.26 (0.32)	0.0369	0.0149	89.82	89.98	14.62	12.93	10.51

**Table 4:** Rate of degradation of binary and ternary mortars of alkali-activated mortars.

Sample	Kf	Remark	kc	Remark
PCM	−0.016	No effect	−0.149	No effect
OPC 2.5	0.333	Influence	−0.107	No effect
OPC 5	0.431	Influence	−0.436	No effect
OPC 10	0.249	Influence	−0.056	No effect
OPC 30	0.375	Influence	0.095	Influence
RHA 25	0.170	Influence	−0.125	No effect
RHA 50	0.033	Influence	−0.011	No effect
RHA 75	0.002	Influence	−0.033	No effect

K value: K = ((Resistance at 28 days − Resistance after cycle)/Resistance at 28 days).

**Table 5:** Reaction degree after paste HCl attack (1:20).

Sample	Insoluble in HCl (%)	Reaction Degree (σ)
Binary		
OPC 0 ^a^	74.51	25.49
OPC 2.5	55.88	44.12
OPC 5	57.66	42.34
OPC 10	44.12	55.88
OPC 30	32.08	67.92
Ternary		
RHA 0	44.12	55.88
RHA 25	50.98	49.02
RHA 50	56.86	43.14
RHA 75	58.82	41.18

^a^ Only with bottom ash as precursor.
